# A Unique Case Presentation of Synchronous Early-Stage Invasive Lobular Carcinoma of the Breast and Marginal Zone Lymphoma of the Ipsilateral Breast

**DOI:** 10.7759/cureus.84227

**Published:** 2025-05-16

**Authors:** Chaewon Hwang, Alvin Krishna, Raj Kavadi, Kara Lynne Leonard

**Affiliations:** 1 Radiation Oncology, Tufts Medical Center, Boston, USA; 2 Radiation Oncology, Beth Israel Deaconess Medical Center, Harvard Medical School, Boston, USA; 3 Radiation Oncology, Brown University Health, Providence, USA

**Keywords:** breast cancer management, breast conservation surgery, invasive lobular carcinoma of the breast, involved site radiation therapy, marginal zone b-cell lymphoma, non-hodgkin lymphoma, whole breast radiotherapy

## Abstract

We report a rare case highlighting the treatment strategy and literature review of synchronous, early-stage ipsilateral breast invasive lobular carcinoma (ILC) and marginal zone lymphoma (MZL). A 78-year-old woman presented with mammographically detected masses in the right breast. Diagnostic mammography revealed two distinct lesions: one at the 10 o’clock position measuring 1 cm, confirmed by biopsy as MZL, and another at the one o’clock position measuring 2.3 cm, diagnosed as estrogen receptor (ER)/progesterone receptor (PR) positive, HER2-negative ILC.

Staging PET imaging revealed no evidence of locoregional or distant metastatic disease. The lymphoma was staged as stage IAE, and the ILC was staged as stage IA (pT1a, pN0, cM0, G1). A breast-conserving treatment approach was selected, consisting of lumpectomy and involved site radiation therapy (ISRT) to the right breast. Given the synchronous presentation of both malignancies within the same breast, whole breast irradiation was delivered using the UK Standardisation of Breast Radiotherapy (START) fractionation regimen (40.05 Gy in 15 fractions), allowing adequate dosing for both ILC and MZL. While standard dosing for breast-based MZL typically ranges from 24 to 30 Gy, the treatment plan was adjusted to address the synchronous ILC.

At five-month follow-up, the patient showed no clinical or radiographic evidence of disease on surveillance mammography. She remained without clinical signs of MZL recurrence in accordance with the National Comprehensive Cancer Network (NCCN) guidelines.

A review of the literature identified a case series involving 37 patients with synchronous breast carcinoma and non-Hodgkin lymphoma, among whom 5.4% had MZL and 10.8% had ILC. Only one patient in the series was reported to have both ILC and MZL, with the MZL staged as IV. Typically, the second malignancy is diagnosed after treatment of the first, resulting in sequential management. In contrast, our case is distinctive in that both cancers were discovered concurrently and at an early stage within the same breast, enabling simultaneous and coordinated treatment.

This case suggests that in rare instances of synchronous early-stage breast cancer and lymphoma, a combined approach utilizing breast-conserving surgery and ISRT may offer an effective and streamlined treatment paradigm.

## Introduction

The American Cancer Society estimates that 310,720 new cases of invasive breast cancer will be diagnosed in women in 2024, with approximately 10% of these being invasive lobular carcinoma (ILC) [1,2]. Additionally, around 80,620 individuals are expected to be diagnosed with non-Hodgkin lymphoma (NHL), with marginal zone lymphomas (MZLs) typically accounting for 5-10% of all lymphoma cases [3,4]. Given these statistics, the occurrence of synchronous breast cancer and lymphoma is quite rare, underscoring the unique nature of such cases.

There are generally two broad categories of lymphoma of the breast: primary breast lymphoma and secondary breast lymphoma. Secondary breast lymphoma is one that develops from a site other than the breast initially, and eventually spreads to the breast. For lymphoma to be categorized as primary breast lymphoma, the following criteria have been accepted in literature: (a) both mammary tissue and lymphomatous infiltrate present in close association in an adequate specimen; (b) no evidence of widespread lymphoma by standard staging techniques or preceding extramammary lymphoma, although ipsilateral axillary node involvement is allowed if both lesions are present simultaneously [5,6].

Risk factors for synchronous primary malignancies include, but are not limited to, environmental exposures, lifestyle factors, and host-related components. For instance, mutations in BRCA1/BRCA2 or the use of hormone therapy can increase the risk of secondary malignancies, such as ovarian and endometrial cancers, in breast cancer patients [7].

Not surprisingly, there is a lack of data on the optimal treatment regimen for patients with synchronous breast cancer and lymphoma, particularly when these tumors occur within the same anatomical region. Current treatment recommendation for NHL varies according to the different subtypes of NHL. For MZL, involved site radiation therapy (ISRT) is preferred, with the radiation treatment dose being 24-30 Gy in 2 Gy per fraction to the involved organ or compartment, per the International Lymphoma Radiation Oncology Group (ILROG) and the National Comprehensive Cancer Network (NCCN) guidelines [8,9]. For breast cancer treatment, regarding adjuvant radiation therapy (RT) following breast conservation surgery, different treatment approaches are possible, including partial breast irradiation and whole breast irradiation. One of the most common treatment paradigms includes whole breast RT, 40.05 Gy/15 fx, per the NCCN guidelines [10].

In this case, we present our experience treating a patient with synchronous stage IAE non-Hodgkin low-grade B-cell lymphoma, of marginal zone origin, and stage IA (pT1a, pN0, cM0, G1, estrogen receptor (ER)+, progesterone receptor (PR)-, HER2-) ILC, both located within the right breast.

## Case presentation

We present a 78-year-old female with no family history of breast cancer, who initially presented with an abnormal screening mammogram. She did not self-palpate any abnormal breast nodules, and she reported no breast discharges, breast erythema, or edema. She did not have night sweats, chills, fever, or weight loss in the past six months. A bilateral diagnostic mammogram with ultrasound revealed a right one o'clock, 4 cm from nipple, irregular, hypoechoic solid nodule, measuring 0.3 x 0.6 x 0.4 cm, and a right 10 o'clock, 8 cm from nipple, irregular shadowing mass corresponding to a new oval dense appearing lesion in the upper aspect of the breast and a right 11 o'clock, 7 cm from nipple, smoothly contoured, hypoechoic nodule versus complex cyst, measuring 0.3 x 0.3 x 0.2 cm.

A core biopsy of the one o'clock lesion showed non-Hodgkin low-grade B-cell lymphoproliferative disorder favoring marginal zone lymphoma. The immunohistochemistry (IHC) panel was diffusely positive for CD20, Bcl-2, and partial CD43. Pertinent negative markers included CD10, Bcl-6, CD23, Bcl-1, and CD5. The Ki-67 immunostaining highlighted a low-proliferation index overall of <10%. Polymerase chain reaction (PCR) was positive for clonal IGH rearrangement (IgH-FR1, IgH-FR2, and IgH-FR3). Fluorescence in situ hybridization (FISH) was negative for MALT1 rearrangement.

**Figure 1:**
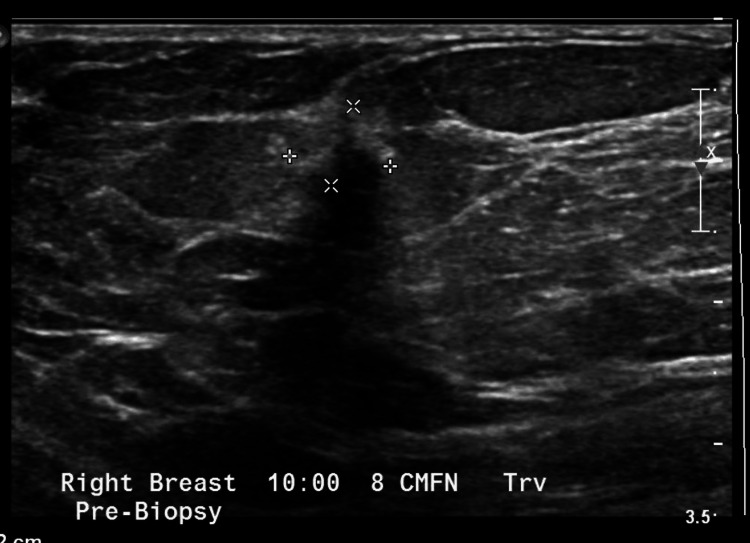
Ultrasound of the right breast. Ultrasound of the right breast further characterized the 10 o'clock lesion that was biopsy-proven to be invasive lobular carcinoma. The borders of the lesion are defined by "x".

**Figure 2:**
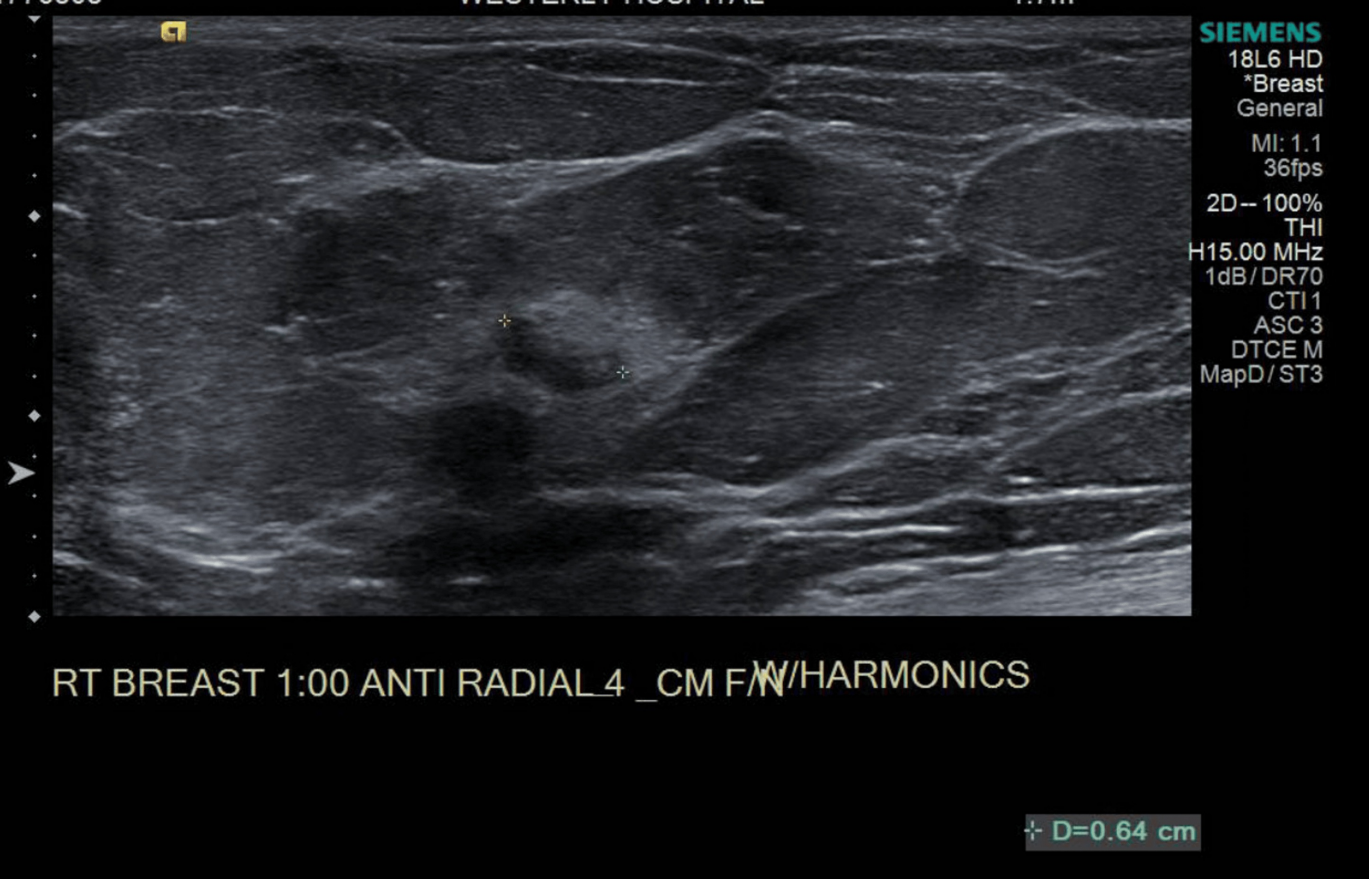
Ultrasound of the right breast for the one o'clock lesion. This ultrasound further delineates the one o'clock lesion in the right breast, which was biopsy-proven to be marginal zone lymphoma. The crosshairs give an approximate dimension.

**Figure 3:**
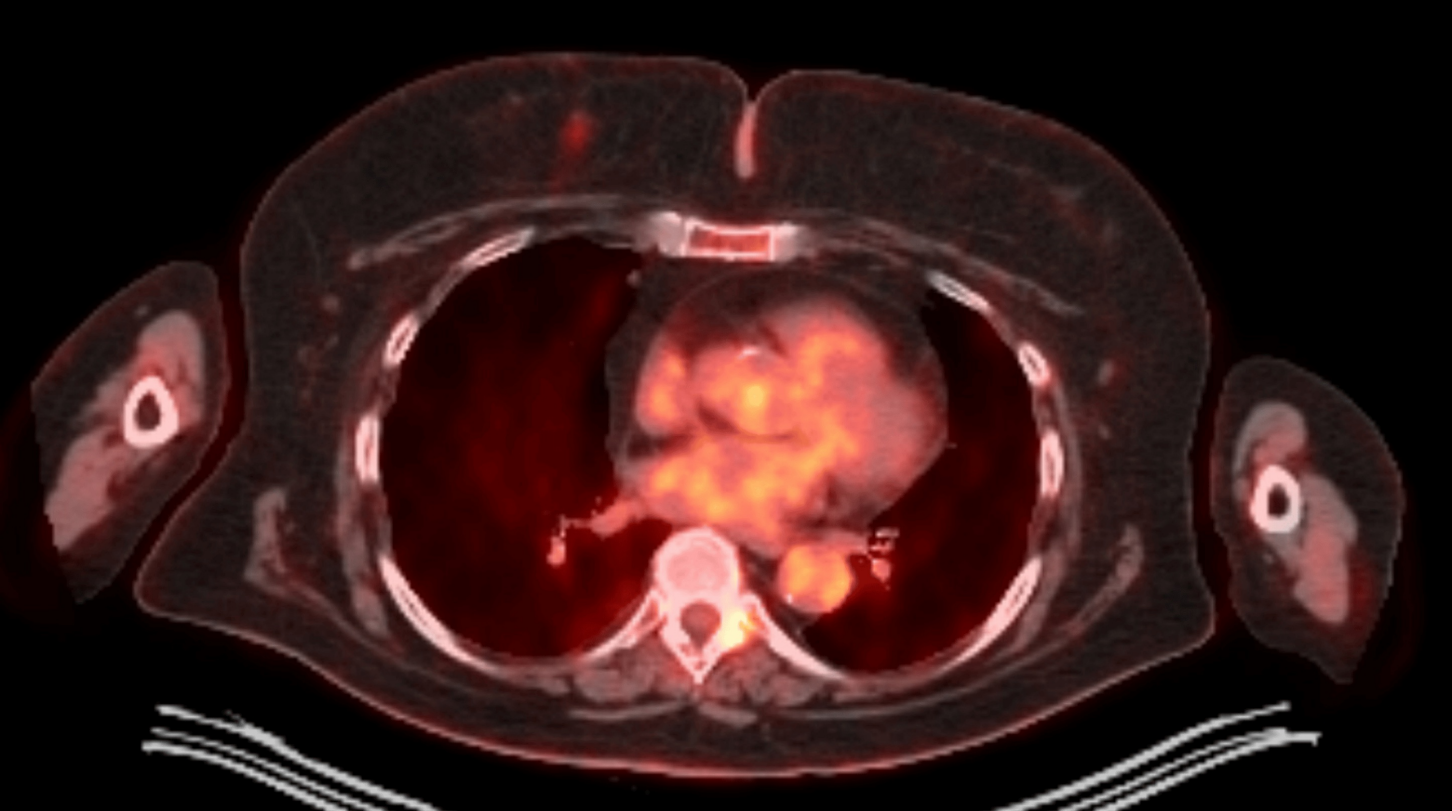
PET-CT showing axial view of the breast, axillary, and thoracic area. PET demonstrates an avid lesion at the one o'clock position in the right breast. There are no PET-avid lymph nodes in the axilla. This one o'clock lesion in the breast was biopsy-proven to be marginal zone lymphoma.

**Figure 4:**
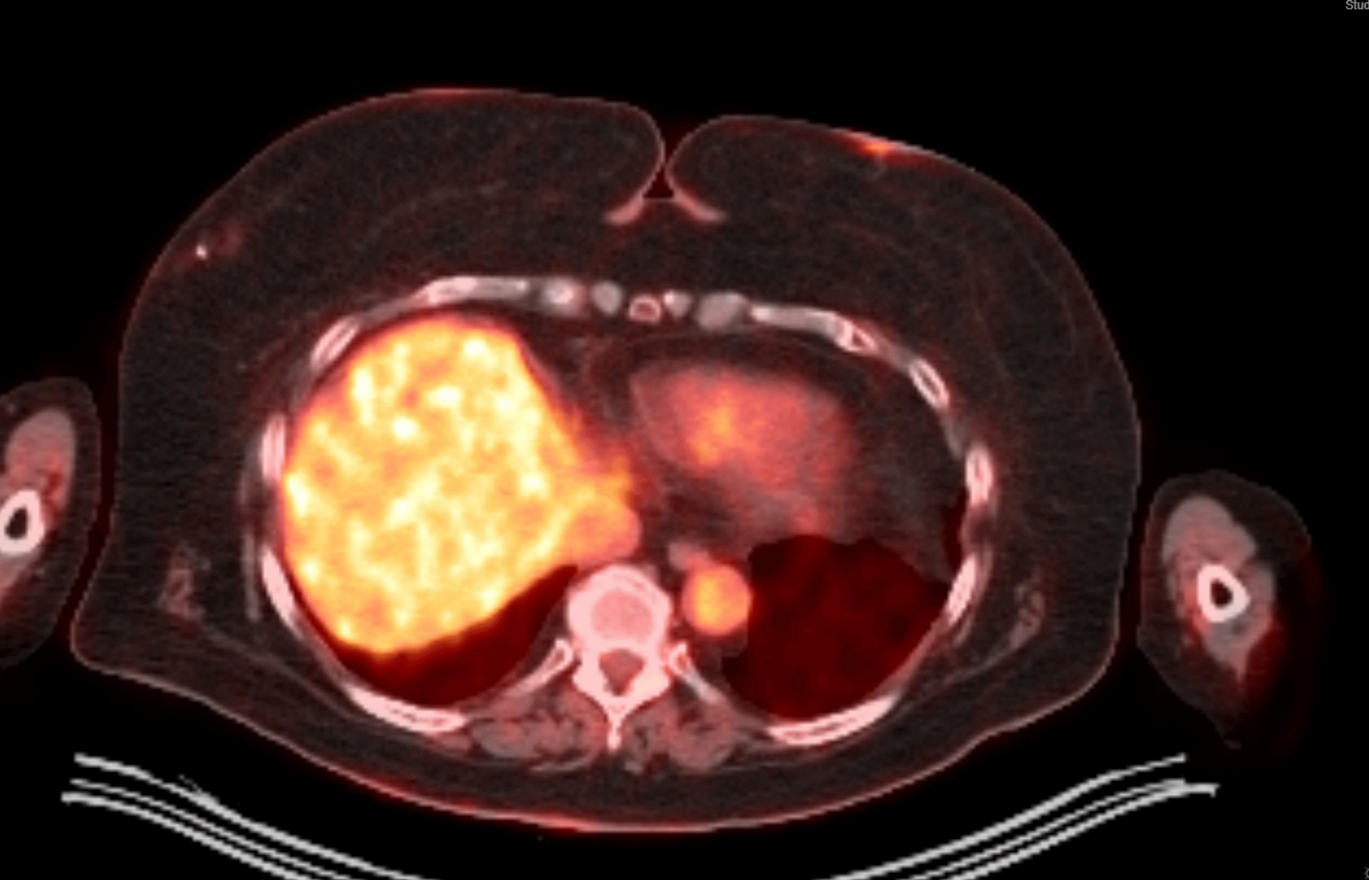
PET-CT (axial view) showing slightly more inferior view compared to Figure 1. PET showing an avid lesion at the 10 o'clock position in the right breast that was biopsied, with a biopsy clip in place. This was biopsy-proven to be invasive lobular carcinoma of the breast.

An ultrasound-guided biopsy of the 10 o'clock lesion (Figure 1) initially demonstrated fibrofatty breast parenchyma and lymphoid tissue, although a re-biopsy was done, which demonstrated infiltrating lobular carcinoma (ILC), Nottingham grade 1, estrogen receptor (ER) 95% positive, progesterone receptor (PR) negative, and HER2/neu negative. Ultrasound of the right breast also demonstrated the one o'clock lesion (Figure 2). A bilateral breast MRI showed biopsy-proven right breast invasive lobular carcinoma at 10 o'clock, measuring 2.3 cm, and a biopsy-proven right breast lymphoma at one o'clock, measuring 1.0 cm. A PET-CT was done showing a biopsy clip of the lateral right breast without discrete/focal fluorodeoxyglucose (FDG) uptake and no convincing evidence of FDG-avid locoregional or distant disease (Figures 3, 4). The final stage for the marginal zone lymphoma was stage IAE, as this was a single extranodal lesion without nodal involvement and B symptoms.

Following a multi-disciplinary breast cancer tumor board meeting and shared decision-making discussion with the patient regarding the risks and benefits of treatment approaches, including mastectomy and breast-conserving therapy for both cancers, the patient proceeded with segmental mastectomy with sentinel lymph node biopsy for the ILC. This surgery confirmed a 6 mm stage IA (pT1b, pN0 (sn), cM0), Nottingham grade 1, ER 95% positive, PR negative, HER2/neu negative, ILC without lymphovascular invasion. The closest anterior margin was 8 mm, and the other margins were >10 mm. Two sentinel lymph nodes were excised and were negative for metastatic carcinoma.

Radiation therapy planning and treatment

**Figure 5:**
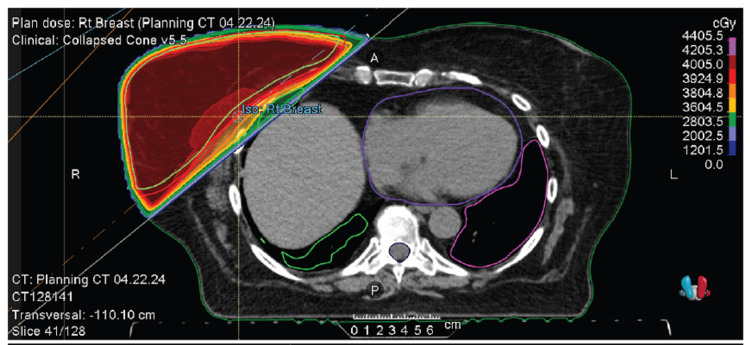
Right whole breast radiation therapy using tangent fields (axial view). The patient was treated with 40.05 Gy in 15 fractions, without a boost.

**Figure 6:**
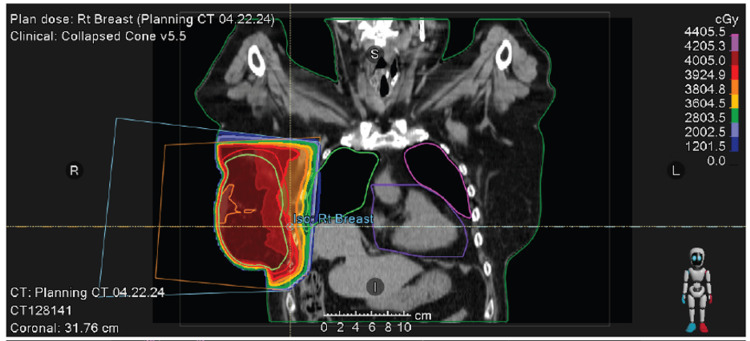
Right whole breast radiation treatment using tangent fields (coronal view).

**Figure 7:**
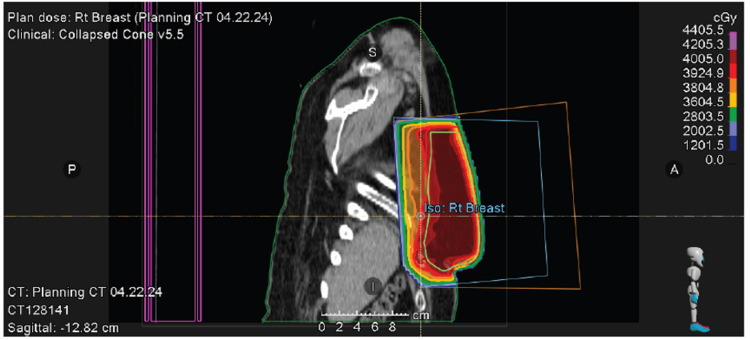
Right whole breast radiation treatment (sagittal view).

CT simulation was performed with the patient in the supine position with arms up. The right whole breast radiation therapy was planned, using opposed tangential fields. The right whole breast was contoured, labeled as Breast_R_CTV, and cropped 5 mm from the skin. The planning target volume (PTV) was created using a uniform 5 mm expansion, cropped 5 mm from the skin. The tumor bed was contoured, and the right breast lymphoma was contoured. Photon beams of 6 MV and 15 MV were used (Figures 5-7). The UK Standardisation of Breast Radiotherapy (START) fractionation (40.05 Gy/15 fractions) was the prescription dose. Although 24-30 Gy is the recommended dosing to treat MZL within the breast, due to the synchronous breast cancer on the ipsilateral breast, a dose of 40.05 Gy in 15 fractions per UK START fractionation was used to treat both forms of cancer, which was reviewed at the breast tumor board conference.

Standard dose constraints for breast treatment were used as below, and all constraints and target goals were met as shown below in Tables 1, 2.

**Table 1:** Dose constraints for treatment of the right breast following breast conservation surgery. The table shows the dose constraints, which include the heart, contralateral lung, and bilateral lungs.

Organ	Dose constraint
Bilateral lungs	V5 Gy < 50%
Heart	V10 Gy < 5%
Heart	V2 Gy < 30%
Heart	Max dose < 1 Gy
Right lung	V12 Gy <15%

**Table 2:** Treatment goals for the treatment of the right whole breast, which includes the right breast tumor bed and right breast lymphoma. The table shows target goals applied to the right whole breast, right tumor bed, and right breast lymphoma. The tumor bed and lymphoma within the breast were separately contoured to ensure proper coverage. CTV: clinical target volume.

Treatment site	Goal
Right breast CTV	V38.048 Gy > 95%
Right breast CTV	Dmax < 42.854 Gy
Right breast CTV	V42.053 Gy < 5%
CTV right breast tumor bed	V40.05 Gy ≥ 95%
CTV right breast lymphoma	V40.05 Gy ≥ 100%

Patient outcomes

During treatment, the patient developed grade 1 dermatitis of the right breast that responded well to topical mometasone. At one-month follow-up, the right breast erythema had improved. The patient did develop a cough during treatment, which was unlikely related to radiation, given V12 Gy of ipsilateral lung was 5%, well below the dose constraint for ipsilateral lung. However, a CT angiogram of the chest was ordered to evaluate for any lung changes. There was no evidence of pulmonary embolism, and no signs of pneumonia or radiation pneumonitis.

The patient did not have clinical or radiographic evidence of disease on follow-up mammogram, which was obtained five months after treatment. She was without clinical evidence of recurrence of lymphoma on the ipsilateral breast on follow-up, per the NCCN guidelines [9,10].

## Discussion

We report an unusual case presentation of early-stage right breast ILC with synchronous right breast extranodal MZL. In a study that specifically looked at the incidence of synchronous breast cancer with NHL, there were 37 cases of synchronous breast cancer with NHL of all types. Of the 37 cases, 5.4% were MZL and 10.8% were ILC cases, with only one person having MZL and ILC, but the stage of MZL for that patient was stage IV [11]. Often, the second tumor is discovered after initiation of the first treatment; therefore, treatment occurs in a stepwise fashion, whether that is treating breast cancer first and then addressing the lymphoma, or vice versa.

Additionally, primary breast lymphomas (PBLs) represent 0.38-0.70% of all NHLs, 1.7-2.2% of all extranodal NHLs, and only 0.04-0.5% of all breast cancer [12]. Therefore, it adds to the rarity of this case that there was synchronous ipsilateral early-stage ILC and MZL of the right breast.

Per the ILROG guidelines, recommendations have been made regarding ISRT to treat various types of lymphomas. ISRT involves radiologically evident disease sites plus an expansion to encompass potential adjacent microscopic disease sites. For localized extranodal MZL, such as the breast, the gross tumor volume (GTV) includes the PET-positive lesion, and the clinical target volume (CTV) includes the entire involved organ or compartment, such as the breast. Involved adjuvant lymph nodes should be included in the CTV. Currently, 24-30 Gy is the standard for MZL [13]. However, due to the synchronous nature of the malignancies in our patient, the UK START fractionation was used to treat both the ILC and lymphoma definitively.

There have been a few phase III randomized clinical trials that have shown dose-intensification was non-inferior to higher doses of RT to treat NHL [13,14]. For example, Lowry et al. has shown in a phase III randomized clinical trial that with a median of 5.6 years of follow-up that there was no difference in overall survival, within-radiation field progression, and progression-free survival between the two treatment arms: 40-45 Gy in 20-23 fractions vs. 24 Gy in 12 fractions [13]. There have been even lower doses, 4 Gy/2 fractions, that were tested in treating follicular or MZL, although longer-term data have revealed 24 Gy/12 fx may have better local control at five years per FoRT trial [14]. However, because we had to consider treatment of both the ILC (breast cancer) and the MZL, with curative intent, we opted for 40.05 Gy/15 fx, as this is the dose used to treat invasive breast cancer following breast conservation surgery.

Although our patient was without disease progression on short-term follow-up at five months, longer-term follow-up per the NCCN guidelines is warranted to evaluate for any recurrence and to evaluate for long-term side effects. The phase III randomized clinical trial has shown that there was lower incidence of acute toxicity, including dry and moist desquamation as well as erythema in the lower dose group (24 Gy/12 fx), but there was no difference in late toxicity, such as skin fibrosis, between the two treatment arms [13]. Our patient did develop acute grade 1 dermatitis of the right breast that responded well to mometasone, and did not have other acute reactions; she tolerated the RT overall well. However, we will continue to follow up with the patient for other long-term side effects such as skin fibrosis.

In our case, because both malignancies were discovered simultaneously at an early stage, our treatment strategy was to treat both simultaneously, first with breast conservation surgery to address ILC, and then with adjuvant whole breast RT to address ILC and MZL.

## Conclusions

The treatment of synchronous malignancies presents a unique challenge in delivering optimal care while minimizing the risk of unnecessary side effects. In the case of synchronous early-stage breast cancer and lymphoma involving the ipsilateral breast, a combined approach of breast conservation therapy and involved site radiation therapy may be an effective treatment paradigm to treat both cancers.
